# A rectovaginal fistula after treatment with bevacizumab. A dangerous side effect needing emergency treatment

**DOI:** 10.1002/ccr3.523

**Published:** 2016-02-26

**Authors:** Michail Galanopoulos, Spyridon Ladias, Xanthippi Tzannetakou, Athanasios Tsigaridas, Alexandros Sarafis, Christos Liatsos, Evangelos Kalafatis

**Affiliations:** ^1^ Department of Gastroenterology“401” General Military Hospital of AthensAthensGreece; ^2^ Department of GastroenterologyGeneral Hospital of Athens “Evaggelismos”AthensGreece; ^3^ 1st Internal Medicine Department401 Army General Hospital of AthensAthensGreece; ^4^ Department of Plastic SurgeryPapageorgiou General Hospital of ThessalonikiThessalonikiGreece

**Keywords:** Bevacizumab, Over‐The‐Scope Clip, rectovaginal fistula

## Abstract

Despite its effectiveness in the treatment of malignant tumors, bevacizumab is associated with a variety of side effects such as the formation of fistulas (i.e., tracheoesophageal, colovaginal, or rectovaginal). It is important to recognize immediately the emergence of this entity in order to discontinue bevacizumab permanently and treat the fistula.

## Question

A 45‐year‐old female patient, who had breast cancer with metastases in the liver and lung, and treated with bevacizumab and docetaxel 5 months ago, reported the passage of flatus and stool through the vagina for about 2 months now. Apart from the breast cancer, her medical history was free and no reference to irradiation or other inflammatory condition in gastrointestinal tract (according to colonoscopy which was done at the time of diagnosis of breast cancer) was mentioned.

## Diagnosis

### A rectovaginal fistula

**Figure 1:**
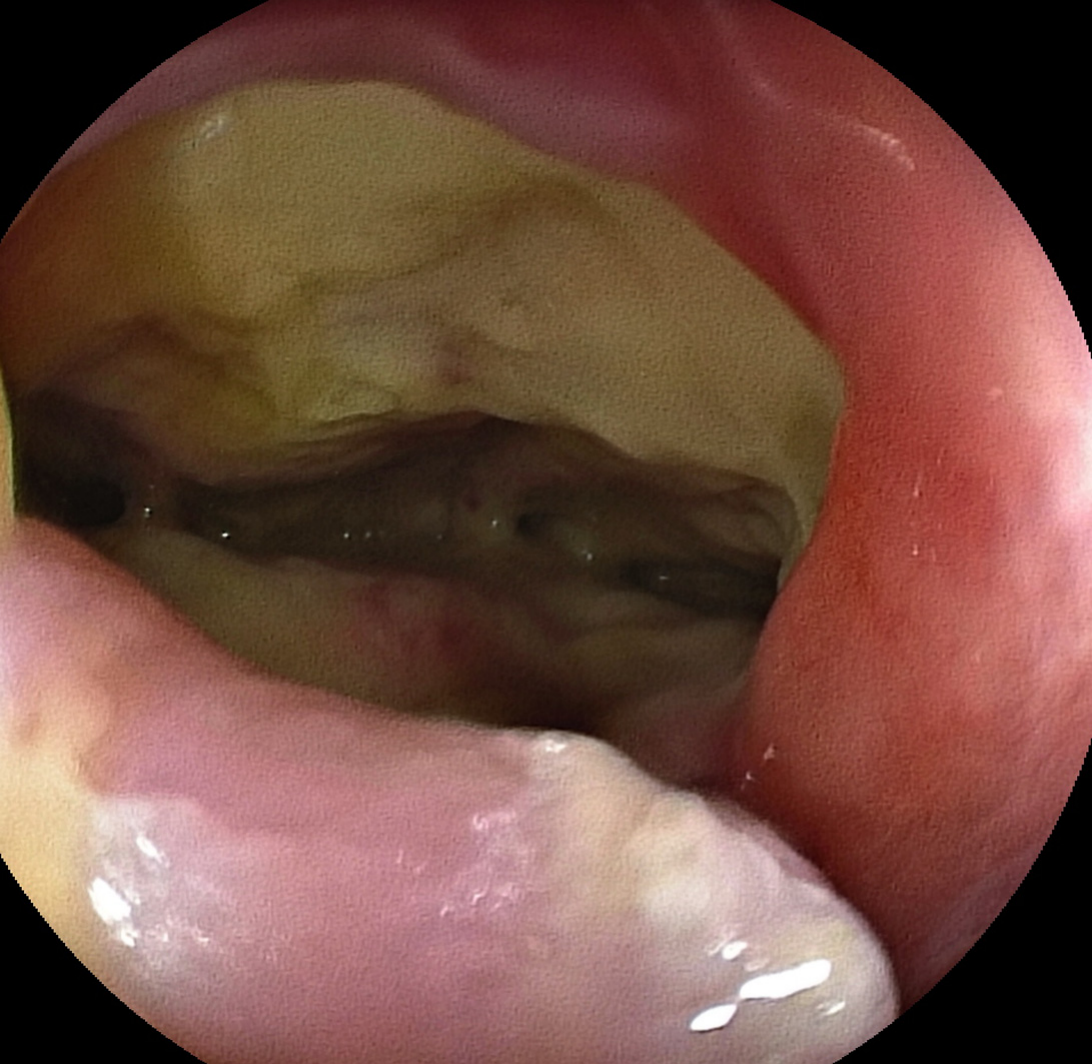
The colonoscopy revealed a rectovaginal fistula on the rectum 3 cm up from anal verge.

**Figure 2:**
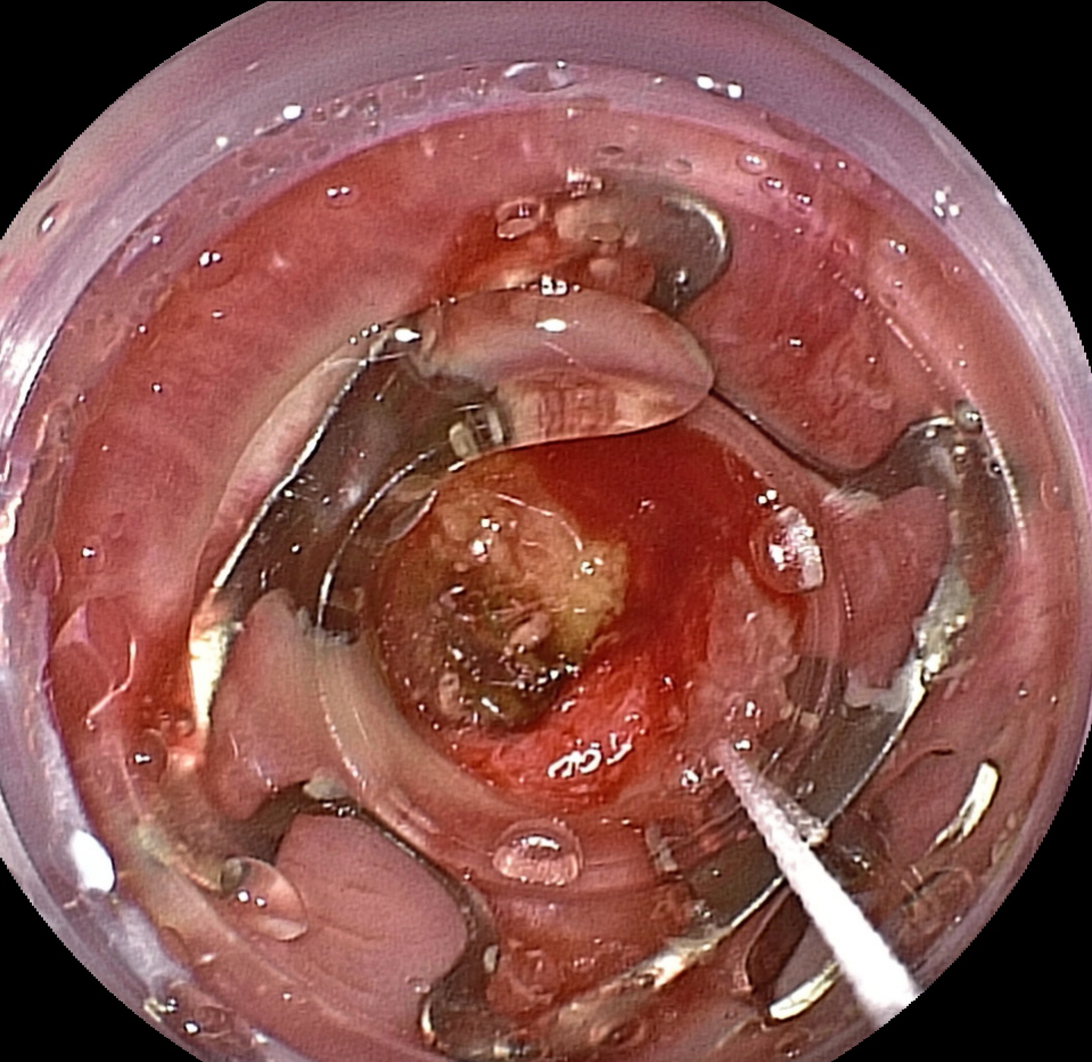
Over‐the‐scope clip with its cap placed around the fistula's edges before vacuum‐assisted closure of fistula.

In colonoscopy was found a rectovaginal fistula on the rectum 3 cm up from anal verge (Fig. 1). An Over‐The‐Scope Clip (Fig. 2) was placed successfully and the occlusion of rectovaginal leak was performed without any complications. The patient had an uncomplicated postoperative recovery.

Despite its effectiveness in the treatment of malignant tumors, bevacizumab, as an angiogenesis inhibitor, is associated with a variety of side effects such as proteinuria, thromboembolic events, and formation of fistulas (i.e., tracheoesophageal, colovaginal, or rectovaginal) 1, 2, 3. According to some clinical trials phase III, bevacizumab has not shown any serious interaction when combined with other chemotherapy regimen and based on AVADO trial the combination with docetaxel has no additional complications 4. However, bevacizumab should be discontinued permanently if bowel fistula or perforation occurs, as happened in our case. Physicians should be aware of this entity so they can act immediately not only by stopping the drug to be prepared for a possible emergence of this side effect later.

In conclusion, herein authors believe that this article prompts the awareness that physician should have, for this relatively uncommon complication of bevacizumab usage. Furthermore, the use of Over‐The‐Scope Clip could be applied more often to other types of fistulas as a therapeutic approach. More studies should be done on producing predictive tools to recognize the patients at risk for bevacizumab‐induced bowel perforation.

## Conflicts of Interests

The authors declare that they have no conflict of interest.

## Informed Consent

Informed consent was obtained from all individual participants included in the study.

## Statement of Human and Animal Rights

All procedures performed in studies involving human participants were in accordance with the ethical standards of the institutional and/or national research committee and with the 1964 Helsinki declaration and its later amendments or comparable ethical standards. This article does not contain any studies with human and animals performed by any of the authors.
